# Using machine learning to identify patient characteristics to predict mortality of in-patients with COVID-19 in South Florida

**DOI:** 10.3389/fdgth.2023.1193467

**Published:** 2023-07-28

**Authors:** Debarshi Datta, Safiya George Dalmida, Laurie Martinez, David Newman, Javad Hashemi, Taghi M. Khoshgoftaar, Connor Shorten, Candice Sareli, Paula Eckardt

**Affiliations:** ^1^ Christine E. Lynn College of Nursing, Florida Atlantic University, Boca Raton, FL, United States; ^2^ College of Engineering & Computer Science, Florida Atlantic University, Boca Raton, FL, United States; ^3^ Memorial Healthcare System, Hollywood, FL, United States

**Keywords:** COVID-19 pandemic, random forest classifier, gini index, feature analysis and prediction, SHAP (Shapley additive explanation), SMOTE (Synthetic minority over-sampling techniques), AI/ML, caring data science

## Abstract

**Introduction:**

The SARS-CoV-2 (COVID-19) pandemic has created substantial health and economic burdens in the US and worldwide. As new variants continuously emerge, predicting critical clinical events in the context of relevant individual risks is a promising option for reducing the overall burden of COVID-19. This study aims to train an AI-driven decision support system that helps build a model to understand the most important features that predict the “mortality” of patients hospitalized with COVID-19.

**Methods:**

We conducted a retrospective analysis of “5,371” patients hospitalized for COVID-19-related symptoms from the South Florida Memorial Health Care System between March 14th, 2020, and January 16th, 2021. A data set comprising patients’ sociodemographic characteristics, pre-existing health information, and medication was analyzed. We trained Random Forest classifier to predict “mortality” for patients hospitalized with COVID-19.

**Results:**

Based on the interpretability of the model, age emerged as the primary predictor of “mortality”, followed by diarrhea, diabetes, hypertension, BMI, early stages of kidney disease, smoking status, sex, pneumonia, and race in descending order of importance. Notably, individuals aged over 65 years (referred to as “older adults”), males, Whites, Hispanics, and current smokers were identified as being at higher risk of death. Additionally, BMI, specifically in the overweight and obese categories, significantly predicted “mortality”. These findings indicated that the model effectively learned from various categories, such as patients' sociodemographic characteristics, pre-hospital comorbidities, and medications, with a predominant focus on characterizing pre-hospital comorbidities. Consequently, the model demonstrated the ability to predict “mortality” with transparency and reliability.

**Conclusion:**

AI can potentially provide healthcare workers with the ability to stratify patients and streamline optimal care solutions when time is of the essence and resources are limited. This work sets the platform for future work that forecasts patient responses to treatments at various levels of disease severity and assesses health disparities and patient conditions that promote improved health care in a broader context. This study contributed to one of the first predictive analyses applying AI/ML techniques to COVID-19 data using a vast sample from South Florida.

## 1. Introduction

As of March 2023, the United States has the highest number of global cumulative SARS-CoV-2 (COVID-19) cases reported at over 100 million and is ranked first for COVID-19 cumulative deaths at over 1 million (1). Florida alone accounts for over 7.5 million confirmed COVID-19 cases and over 88,000 deaths (2). To date, there appears to be a dearth of information published about COVID-19 in South Florida that describes COVID-19 patient characteristics and identifies and validates factors that predict COVID-19 disease progression and “mortality.” This is one of the earlier studies that can be used to predict “mortality” and understand features important to patients hospitalized with COVID-19 who are at risk of dying.

The overwhelming influx of COVID-19-infected patients with severe illness continues to cause unprecedented clinical and direct medical cost burdens. Early prediction of the “mortality” of patients hospitalized with COVID-19 can improve patient outcomes and decrease death rates by guiding treatment plans and assuring efficient resource allocation (3). There is a need to investigate factors associated with poor prognosis for patients in South Florida to assist in identifying patients with COVID-19 who are at higher risk of severe illness and subsequent “mortality.” Identifying such factors is pivotal in monitoring disease progression and targeting individualized interventions. Predicting critical clinical events in the context of relevant individual risks is a promising option for guiding clinical care, optimizing patient outcomes, and allocating scarce resources to reduce “mortality” with a subsequent decline in the overall COVID-19 burden.

Clinical care largely depends on manifestations of COVID-19 symptomology. The diverse clinical spectrum of COVID-19 ranges from asymptomatic presentation to severe acute respiratory syndrome and death. Common classifications of COVID-19 illness are mild (i.e., no pneumonia), severe (i.e., dyspnea, oxygen saturation ≤93%, the proportion of arterial partial pressure of oxygen to fraction of inspired oxygen <300 mm Hg), and critical (i.e., respiratory failure, multiple organ dysfunction) (4). Cumulative evidence indicates that manifestation and the prognosis for severe disease are associated with comorbidities, age, and sex (5–8). Chronic comorbidities posing a significant risk for severe disease progression appear to include cancer, heart failure, coronary artery disease, kidney disease, chronic obstructive pulmonary disease, obesity, sickle cell anemia, and diabetes mellitus (9–13). Extant literature indicates that individuals ≥60 years with comorbidities are at greater risk of severe disease and subsequent death, with “mortality” being highest among individuals ≥70 years, regardless of comorbidities (10, 14). Further, males consistently have a higher “mortality” rate when compared to females, regardless of preexisting comorbidities or age group (15, 16).

Direct medical costs associated with COVID-19 treatment are primarily associated with the severity of the disease (17, 18). Evidence indicates that the cost of care for patients with severe symptomology has higher direct costs than those with less severe infections (19). Moreover, higher costs appear to be driven by the increased use of hospital resources and a higher risk of “mortality” (17, 19, 20). Literature suggests that predictive modeling can optimize the future treatment of patients hospitalized with COVID-19 by guiding early time-sensitive clinical interventions that improve the quality of care, decrease “mortality”, and rationalize resource allocation (21, 22).

### 1.1. COVID-19 mortality and machine learning

According to the extant literature, numerous retrospective analyses have investigated the statistical significance of various features in relation to COVID-19 “mortality”. These features include demographics such as race/ethnicity, age, gender, BMI, as well as comorbidities like diabetes, hypertension, COPD, among others (23). Many of these demographics and comorbidities have emerged as key determinants of COVID-19-related “mortality”.

Similarly, several ML-based models have been developed to predict COVID-19-related “mortality” by considering the severity of the disease (22, 24, 25). For example, Kirby et al. 2021 (24), created a logistic regression model to forecast disease severity and “mortality”. The severity score was derived from a literature survey of COVID-19 patients, categorizing them based on comorbidity conditions, ICU admissions, and the need for mechanical ventilation, among other factors. Multivariate logistic regression was utilized to classify patients into four severity categories using the newly adopted “COVID-related high-risk Chronic Conditions” (CCCs) scale (24), with the model achieving an accuracy of approximately 66%. It is worth mentioning that the CCC scale was manually constructed, assigned scores to individual patients, and the model incorporated other statistical patient information to predict these scores. Based on statistical analysis, the study concluded that age and gender served as significant risk predictors for COVID-19 disease severity. However, the study did not examine the model's interpretability for its decision-making process.

In a more detailed predictive analysis conducted by Zhu et al. 2020 (26), a deep learning approach was employed to identify key factors from a pool of 181 data points in a multicenter study. The identification of important features involved permuting different feature values and observing the impact on model performance. By determining the top 5 most important features, the study trained a new model to predict “mortality,” which yielded higher accuracy for the top 5 important features. However, it is important to note that this analysis presented potential limitations as it relied on sophisticated lab biomarkers—such as O2 Index, D-dimer, and neutrophil-lymphocyte count—which may not be universally available and thus should not serve as a common platform for understanding risk factors.

In another study by Zhao et al. 2020 (22), a logistic regression analysis was utilized to classify ICU admission and “mortality” among COVID-19 patients. The researchers achieved improved outcomes by incorporating a broader range, rather than specific, of lab biomarkers and patient medical reports from a relatively small cohort of 641 patients. The Area Under the Curve (AUC) scores were reported as 0.74 for predicting ICU admission and 0.83 for “mortality.” However, it is important to consider the limitations of the study's small cohort size. The observed success of the model could potentially be attributed to overfitting and may not be generalizable to a larger population.

In the realm of retrospective analysis regarding “mortality” in COVID-19 patients, Kirby et al. 2021 (24), investigated the performance of various models in predicting “mortality”. The study compared models such as **R**andom **F**orest (RF) classifier, **S**upport **V**ector **M**achine (SVM), logistic regression, and gradient boosting. Notably, the RF emerged as the top-performing model among the others in predicting “mortality”. The RF classifier achieved an accuracy of 79% on the test dataset. However, it is important to consider that the higher accuracy might be influenced by the utilization of a small population and potential overfitting. Furthermore, the investigation delved into determining the most important features indicative of “mortality” risk. Lime-SP (model agnostic) prediction analysis revealed that age and gender were the most important predictors within the specific patient demographics (25). The study also identified **B**lood **U**rea **N**itrogen levels (BUN), creatinine, and neutrophils-lymphocyte levels as predictive of “mortality” risk. Nevertheless, it is noteworthy that this study's dataset comprised 797 data points and was solely focused on predicting “mortality” for ICU patients on their admission day to ICU.

### 1.2. Purpose

This study aims to develop an AI-driven decision support system that effectively predicts “mortality” of patients hospitalized with COVID-19 by identifying the most significant features. It aims to contribute to predictive analyses in applying AI/ML techniques to COVID-19 data, utilizing a substantial sample from South Florida. The study further seeks to facilitate the development of quantitative AI-based techniques by integrating statistics, data science, and machine learning methods. Through this integration, the study aims to explore the variables influencing “mortality” and contribute to the creation of objective, data-driven decision-support systems to enhance disease management practices.

## 2. Materials and methods

### 2.1. Dataset collection and subject information

The study was approved by our respective Institutional Review Board (IRB) with the exemption of informed consent and HIPAA waiver. IRB also determined that this project is exempt from further review. Data were obtained from the South Florida Memorial Health Care System between March 14th, 2020, and January 16th, 2021, and analyzed by the co-authors from Christine E. Lynn College of Nursing and College of Engineering and Computer Science at Florida Atlantic University. For this project, retrospective data on “5,371” patients were confirmed cases of COVID-19 as defined by an RT-PCR assay of nasal and pharyngeal swab specimens, and hospitalized for COVID-19-related symptomology, was collected from the large extensive healthcare system in South Florida. Data initially contained 203 columns (independent variables) which included “patients” sociodemographic characteristics' (e.g., age, sex, BMI, smoking status.), “pre-hospital comorbidities” (e.g., diarrhea, diabetes, pneumonia.), and “medications” (e.g., **A**ngiotensin **R**eceptor **B**lockers (ARBs), **A**ngiotensin **C**onverting **E**nzyme (ACEs) inhibitors).

### 2.2. Study design considerations

**Figure 1:**
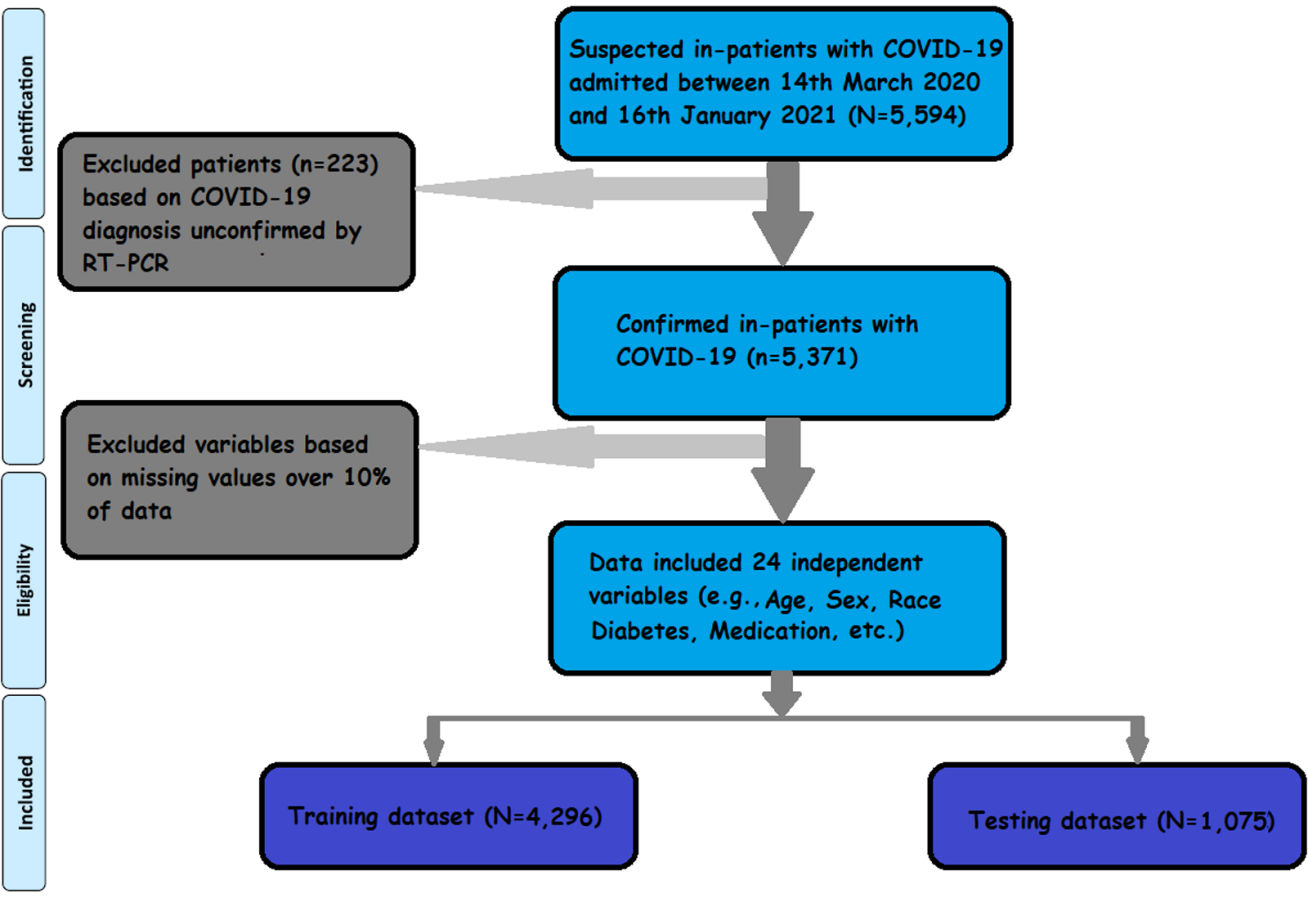
Work flowchart. Flowchart depicts the data inclusion strategy. Of the “5,594” hospitalized cases, “5,371” are confirmed COVID-19 cases. After excluding variables for missing data, 25 variables (among them, 24 are independent variables, and “mortality” is a dependent variable) meet the inclusion criteria.

Figure 1 presents the flowchart outlining the patient selection process. Out of the “5,594” hospitalized patients, “5,371” individuals had confirmed tests for COVID-19-positive. These “5,371,” who were admitted to the hospital with COVID-19, formed the input data set in our analysis. The comparison was made with a subgroup of “615” admitted patients who “expired”, while the remaining “4,765”patients were discharged from the hospital.

To ensure data quality, preprocessing steps were performed. This involved removing independent variables with repeated value entries and excluding attributes with over 10% (27) missing values from the list of potential predictors. As a result, 25 variables, including 24 independent variables and one dependent variable (“mortality”), were utilized for model training.

### 2.3. Data classification

The current work was based on binary classification problems, meaning the dependent variable was segmented into “survival” and “mortality,” which were either classified as “survived” (“0”) or “expired” (“1”) and was mapped to numerical values before being provided for modeling. A classification model sought to draw conclusions from the observed values when the inputs were one or many, and the model predicted the outputs of one or many.

Among the 24 independent variables used in the current research, 2 variables—age, and BMI—were transformed from continuous to categorical. Age was categorized based on age matrices (28) where “younger adults” ranged between 20 and 34, “middle adults” ranged between 35 and 64, and “older adults” ranged between 65 and 90 years. Similarly, BMI was categorized based on the BMI metrics (29), with “underweight” defined as below 18.50, “normal weight” ranged between 18.5 and 24.9, “overweight” ranged between 25 and 29.9, and “obese” defined as 30 or greater.

Additionally, this study employed overwriting the dummy coding rather than relying on computer-generated methods like “one-hot-encoding.” (30) This unique approach leveraged interpretability and domain knowledge while benefiting from the power and efficiency of AI modeling. This approach allowed clinicians to interpret the results (features) more effectively. Although it might introduce a slight bias into the model, potentially impacting its optimal performance (discussed in Section 3.3), it provided clinicians with a better understanding of health status based on the features analyzed. For instance, the study investigated which age group or sex had a more significant impact, whether hypertensive medications (ARBs, ACEIs) were beneficial, or if individuals with diabetes were more vulnerable to “mortality”.

### 2.4. Correlation check

To further understand the efficiency and adequacy of 24 independent variables in characterizing “mortality”, a tetrachoric correlation (31) analysis was performed, and the resultant correlation coefficients of all pairs of variables showed a low correlation (<0.50) except for a correlation between **C**hronic **K**idney **D**isease (CKD, stage 5) and dependence on renal dialysis; they were positively correlated (0.66). Correlation analysis was completed only for exploratory data analysis. However, this research did not use correlation as a guideline for selecting features, as two correlated features can further improve the model accuracy when they are part of the same data set (32).

### 2.5. Data splitting

For performance evaluation, data were divided into training and testing sets. The Scikit-learn library (33) randomly splits the dataset into two input sets. Among the “5,371” patients, 80% (*N* = 4,296) of data were split into a training data set, and the rest, 20% (*N* = 1,075) were split into the test data set. Both “train” and “test” datasets contained 89% from the “survival” class and 11% from the “expired” class.

### 2.6. Resampling data

The training dataset was imbalanced; “3,804” cases were classified as “survived”*,* and “492” were “mortality”. Oversampling was performed to modify uneven datasets to create balance. A method that performs over-sampling is the **S**ynthetic **M**inority **O**ver-sampling **T**echnique (SMOTE), by synthesizing new examples as opposed to duplicating examples (34). The SMOTE was applied to the training data set in the cross-validation to avoid the possibility of over-fitting; however, this technique was not applied to the test data set for model evaluation that prevents data leakage (32).

## 3. Results

### 3.1. Cohort description

Included within the model were “5,371” patient entries (including training and test data) who were COVID-19-positive and 24 variables. Patients' data were categorized into “patients” sociodemographic characteristics' (e.g., age, sex, BMI, smoking status, etc.), “pre-hospital comorbidities” (e.g., diarrhea, diabetes, pneumonia, etc.), and “medications” (e.g., ARBs & ACEs) (See Table 1).

**Table 1:** Parameters and characteristics.

Dependent variables Mortality	Categories Expired Survived	*n* 615 4,756		% 11.45 88.55	
Independent variables	Categories	Expired		Survived	
		*n*	%	*n*	%
Patients’ sociodemographic characteristics					
Age	Young adults	10	0.19	621	11.56
	Middle adults	158	2.94	2,345	43.66
	Older adults	447	8.32	1,790	33.33
Sex	Female	256	4.77	2,400	44.68
	Male	359	6.68	2,356	43.87
Race	Black	158	2.94	1,518	28.26
	Others	325	6.05	2,444	45.50
	White	132	2.46	794	14.78
Ethnicity	Hispanic	220	4.10	1,572	29.27
	Non-Hispanic	395	7.35	3,184	59.28
Smoking status	Never	459	8.55	3,992	74.33
	Former	139	2.59	642	11.95
	Current	17	0.32	122	2.27
Pre-hospital comorbidities					
COPD	No	512	9.53	4,388	81.70
	Yes	103	1.92	368	6.85
Kidney disease (stages 1 to 4)	No	398	7.41	4,112	76.56
	Yes	217	4.04	644	11.99
Kidney disease (stg5)	No	584	10.87	4,606	85.76
	Yes	31	0.58	150	2.79
Diarrhea	No	420	7.82	4,133	76.95
	Yes	195	3.63	623	11.60
Hypertension	No	82	1.53	1,731	32.23
	Yes	533	9.92	3,025	56.32
Diabetes	No	266	4.95	2,931	54.57
	Yes	349	6.50	1,825	33.98
Pneumonia	No	307	5.72	2,858	53.21
	Yes	308	5.73	1,898	35.34
Heart failure	No	462	8.60	4,166	77.56
	Yes	153	2.85	590	10.98
Cardiac arrhythmias	No	484	9.01	4,213	78.44
	Yes	131	2.44	543	10.11
Coronary artery disease	No	439	8.17	4,072	75.81
	Yes	176	3.28	684	12.74
Dependence on renal dialysis	No	585	10.89	4,643	86.45
	Yes	30	0.56	113	2.10
Cerebrovascular disease	No	563	10.48	4,471	83.24
	Yes	52	0.97	285	5.31
BMI	Underweight	16	0.30	59	1.10
	Normal weight	120	2.23	755	14.06
	Overweight	179	3.33	1,379	25.67
	Obesity	267	4.97	2,252	41.93
Liver disease	No	592	11.02	4,661	86.78
	Yes	23	0.43	95	1.77
Asthma	No	600	11.17	4,606	85.76
	Yes	15	0.28	150	2.79
HIV	No	612	11.39	4,703	87.56
	Yes	3	0.06	53	0.99
Cancer	No	556	10.35	4,502	83.82
	Yes	59	1.10	254	4.73
Medications					
ARBs	No	423	7.88	3,555	66.19
	Yes	192	3.57	1,201	22.36
ACEIs	No	364	6.78	3,147	58.59
	Yes	251	4.67	1,609	29.96

### 3.2. Statistical analysis

To estimate the predictive value of the 24 variables on “mortality”, 24 individual binary logistic models were conducted (35). As shown in Table 2, all 24 predictors except for ethnicity and asthma were statistically significant in predicting the likelihood of “mortality”. The highest risk factors were older adults (OR = 15.51) and early stages of CKD (OR = 3.48).

**Table 2:** Individual Chi-squaree results of the 24 demographic features predicting “mortality”.

	No “mortality” (*N* = 4,756)		“Mortality” (*N* = 615)					
	*N*	*%*	*N*	*%*	*χ^2^*	*df*	*p*	*OR*
Age	-	-	-	-	286.3	2	<.001	15.51
Young adult	621	13.1	10	1.6	-	-	-	-
Middle adult	2,345	49.3	158	25.7	-	-	-	-
Older adult	1,790	37.6	447	72.7	-	-	-	-
BMI	-	-	-	-	14.3	3	.002	0.76
Underweight	59	1.2	16	2.6	-	-	-	-
Normal	755	15.9	120	19.5	-	-	-	-
Overweight	1,630	34.3	210	34.1	-	-	-	-
Obese	2,312	48.6	269	43.7	-	-	-	-
Sex (Male)	2,356	49.50	359	58.4	17.0	1	<.001	1.43
Race	-	-	-	-	14.2	2	<.001	1.60
Black	1,518	31.9	158	25.7	-	-	-	-
Other	2,444	51.4	325	52.8	-	-	-	-
White	794	16.7	132	21.5	-	-	-	-
Ethnicity (Not Hispanic)	3,184	66.9	395	64.2	1.8	1	.178	0.89
Smoking status	-	-	-	-	36.8	2	<.001	1.88
Never	3,992	83.9	459	74.6	-	-	-	-
Former	642	13.5	139	22.6	-	-	-	-
Current	122	2.6	17	2.8	-	-	-	-
Diabetes	1,807	0.38	351	0.57	76.3	1	<.001	2.11
Hypertension	3,044	0.64	535	0.87	129.5	1	<.001	3.72
COPD	380	0.08	105	0.17	55.3	1	<.001	2.40
Asthma	143	0.03	12	0.02	0.9	1	.334	0.77
CKD stage 1 to 4	666	0.14	215	0.35	191.3	1	<.001	3.48
CKD stage 5 to ESRD	143	0.03	31	0.05	6.0	1	.015	1.63
Heart failure	571	0.12	154	0.25	71.1	1	<.001	2.34
Cancer	238	0.05	62	0.1	17.9	1	<.001	1.88
Cardiac arrythmias	523	0.11	129	0.21	48.5	1	<.001	2.10
Cerebrovascular disease	285	0.06	49	0.08	5.6	1	.018	1.45
Coronary artery disease	666	0.14	178	0.29	82.1	1	<.001	2.39
Liver disease	95	0.02	25	0.04	7.7	1	.006	1.91
HIV	48	0.01	0	0	2.1	1	.150	0.44
Pneumonia	1,902	0.4	308	0.5	23.3	1	<.001	1.51
ARBs	1,189	0.25	191	0.31	10.1	1	.001	1.34
ACEIs	1,617	0.34	252	0.41	11.7	1	<.001	1.35
Diarrhea	618	0.13	197	0.32	146.1	1	<.001	1.19
Dependence on renal dialysis	95	0.02	31	0.05	13.2	1	<.001	2.11

Using a traditional statistics approach, several options are available for selecting the optimal set of key features to include in the model. One of the more common methods includes both forward and backward stepwise approaches. Of these methods, the backward Wald stepwise binary logistic regression was selected for this study as it was considered more conservative and less likely to introduce false positives to the model (36–38).

To avoid overfitting of the model for our specific sample, a 10-fold cross-validation approach was used with Wald's backward binary logistic regression (39). As can be seen in Table 3, of the initial 24 features, the following 11 were retained by the model: age, sex, smoking status, diabetes, hypertension, CKD stages 1–4, heart failure, pneumonia, ARBs, ACEIs, and diarrhea. These features were statistically significant in predicting “mortality” and accounted for approximately 19% of the variability in the model [*χ^2^(13) = 547.88, p < .001, Nagelkerke R ^2 ^= .19*]. Of these retained variables, age had the largest impact in the multivariate binary logistic regression, with older adults being 7.53 times more likely to die than those younger adults when controlling for all other predictors.

**Table 3:** Predicting “mortality” using backward logistic regression results feature selection.

							95% CI for OR	
	B	SE	Wald	df	*p*	OR	Lower	Upper
Age (young adult)			117.35	2	<.001			
Age (Middle adult)	0.96	0.34	8.19	1	.004	2.62	1.35	5.06
Age (Older adult)	2.02	0.34	36.04	1	<.001	7.53	3.90	14.56
Sex (Male	0.34	0.09	12.83	1	<.001	1.40	1.16	1.68
Smoking status (Never)			6.97	2	.031			
Smoking status (Former)	0.26	0.12	5.27	1	.022	1.30	1.04	1.63
Smoking status (Current)	0.42	0.28	2.22	1	.136	1.52	0.88	2.64
Diabetes	0.35	0.10	13.13	1	<.001	1.42	1.17	1.72
Hypertension	0.60	0.15	16.78	1	<.001	1.82	1.37	2.42
CKD stage 1–4	0.64	0.11	36.33	1	<.001	1.89	1.54	2.33
Heart failure	0.29	0.12	5.89	1	.015	1.33	1.06	1.68
Pneumonia	0.28	0.09	9.53	1	.002	1.33	1.11	1.59
ARBs	−0.36	0.11	11.66	1	<.001	0.70	0.57	0.86
ACEIs	−0.31	0.10	9.41	1	.002	0.74	0.60	0.90
Diarrhea	1.03	0.10	98.66	1	<.001	2.79	2.28	3.41
Constant	−4.74	0.33	206.70	1	<.001	0.01		

### 3.3. Model performance evaluation

**Figure 2:**
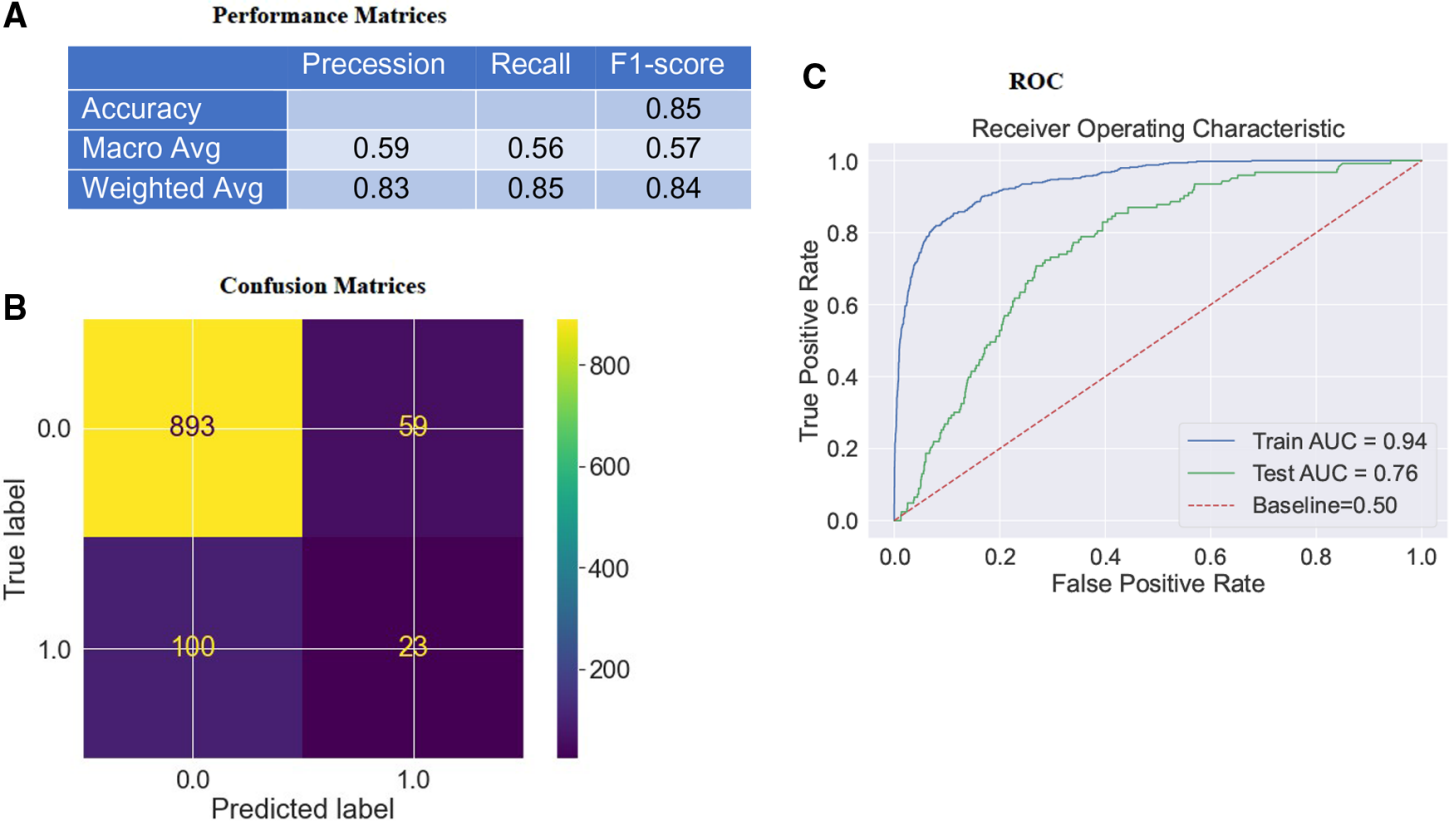
Confusion matrices & AUC curve of the model. (**A**) depicts the model's F1-score (weighted) as 84%. (**B**) depicts that the model accurately classifies “916” instances and misclassifies “159” instances of test data. “Survival” and “mortality”, classifies respectively as “0” or “1”. (**C**) indicates an ROC curve created by comparing TPR and FPR. The **A**rea **U**nder the **R**eceiver **O**perating System (ROC) **C**urve (AUC) for the RF classifier on the testing dataset is about 0.76, which is better than a no-skill classifier with a score of 0.50.

In the previous section, we explored descriptive statistics. The following will assess model accuracy based on the F1-score ([2×precission×recall]/[precission+recall]) (Figure 2A) and **A**rea **U**nder the **C**urve (AUC) (Figure 2C) from RF classifier performance on the test data to interpret the imbalanced data set better.

The AUC scoring system was most appropriate for the imbalanced data set as it accounted for **T**rue **P**ositives (TP), **T**rue **N**egatives (TN), **F**alse **P**ositives (FP), and **F**alse **N**egatives (FN) (40–43). The model accurately classified “916” instances and misclassified “159” test data (Figure 2B). Upon training our RF classifier to distinguish between “survival” and *“*mortality”, we achieved an F1-score (Figure 2A) (weighted) of 84% (Precision: 83% & Recall: 85%) and an AUC (Figure 2C) of 76% in the test data. Consequently, the model demonstrated favorable Bias-Variance and Precision-Recall tradeoffs. It is important to note that the **F**alse **P**ositive **R**ates (FPR) were obtained by subtracting the specificity (**T**rue **N**egative **R**ate; TPR) from “1”, meaning that a lower FPR indicates higher sensitivity (TPR). Therefore, the optimal values for specificity and sensitivity were laid in the top left corner of the ROC curve (Figure 2C).

Challenges encountered in the study included overfitting concerns, dataset pre-treatment, and the model's performance in different classes. We encountered an overfitting issue in our study due to the pre-treatment of the “training dataset.” Specifically, we applied oversampling techniques using SMOTE analysis to address the imbalance in the minority class (“mortality”) while the “test data” remained unaltered. Additionally, to assess the model's performance on an unbiased dataset, the test accuracy was evaluated on a smaller cohort size (*N* = 1,075). However, it is important to note that for the feature importance, **SH**apley **A**dditive ex**P**lanations (SHAP) analysis (discussed in section 4) was conducted on the “training dataset”.

As for the model's performance in different classes, one challenge addressed was the model's inadequate performance in the positive class. This was attributed to training the model using a dataset where 50% of the population experienced expiration (“mortality”), while the remaining 50% were discharged (“survival”) from the hospital. Consequently, the model was optimized for two distinct cohorts: “mortality” and “survival”. Given that the dataset was balanced, both groups held equal importance during the model's training process.

Nevertheless, we observed an imbalance in the model's performance on the new (“test”) dataset. Specifically, the model exhibited good performance in the majority class (“survival”) but relatively poor performance in the minority class (“mortality”). This discrepancy could be attributed to the imbalanced nature of the dataset, where it was easier to predict someone as “survival” than correctly label them as “mortality” due to the predominance of “survival” instances in the test data. To account for this issue, we considered additional metrics to assess the model's performance. These metrics included F1-score, precision, recall, and AUC. Recall represents the ratio of correctly classified positive samples to the total number of positive samples, while precision measures the model's accuracy in predicting the positive class. The F1-score combines precision and recall into a single metric, representing the test's accuracy by taking the harmonic mean of precision and recall. By considering these metrics collectively, we gained a comprehensive understanding of the model's performance.

## 4. Model interpretation

### 4.1. Global feature interpretation

**Figure 3:**
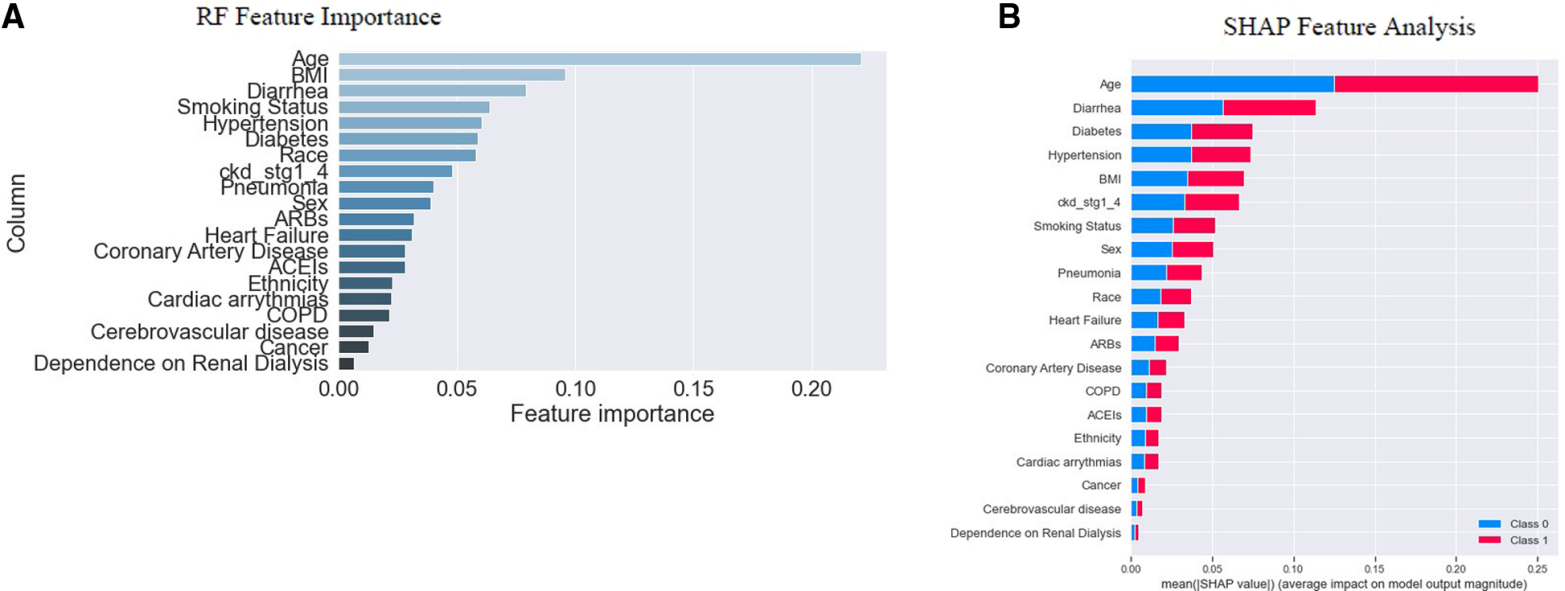
Feature importance. In (**A**), RF's “built-in” method allows us to look at feature importance, corresponding to the decrease in the Gini index of a feature at each split. Notably, the Gini coefficient is used to evaluate the degree of inequality within these features. As the Gini index decreases for a feature, it becomes more important (41, 45). (**B**) indicates the SHAP global feature importance. The important features are in the sequence where the color difference shows the binary classes; 0 (“blue”) indicates “survival”, and “1” (“red”) indicates “mortality”. From the diagrams above, we note that the actual importance of each feature given by the two methods is similar but not identical.

The study established two *post hoc* methods to determine the importance of individual features and their contribution to the model's outcome on the “training dataset”. These methods were based on either the model-based (also known as “built-in”) feature importance (Figure 3A) or SHAP global feature importance method (Figure 3B). Figure 3A displays the importance of each feature analyzed using the model's “built-in” method (41, 44, 45). The model-based feature importance plot highlighted the model's ability to determine classification by the “Gini index,” which measured the inequality among the values of a variable. It established the significance of reducing the “Gini index” in classification. The “Gini index” of a model's input features was summed to “1” (41, 45). The importance was measured by the mean decrease in “Gini impurity,” which could represent the probability of a new sample being incorrectly classified at a given node (weighted by the probability of attaining the node) in a tree, averaged over all trees together in the model (46).

However, a SHAP global importance plot considered each feature's mean absolute value or weights assigned to the model, over all instances of the current dataset (47–50). The SHAP interpretation, being model-agnostic, provided a means to compute feature importance from the model (51). It used Shapley values, based on game theory (52), to estimate how each feature contributed to the prediction (49). By taking the mean absolute value of the SHAP for each feature, we were able to construct a stacked bar plot to visualize importance (Figure 3B). This approach allowed us to focus on feature importance rather than comparing multiple models to determine the most accurate results for the model's best accuracy.

The graph's *x*-axis (Figure 3B) depicted how individual features' SHAP values contributed to predicting a classification problem's outcomes. The features were positioned along the *y*-axis based on their decreasing importance, where a higher position indicated a higher Shapely value or higher risk of “mortality”. The color scheme represented binary variables, with “blue” indicating “survival” and “red” indicating “mortality”. A skewed distribution indicated the greater importance of the feature (45).

The depicted plot (Figure 3B) illustrates the significance of each feature in relation to “mortality”. After analyzing individual “feature inputs” contribution to the model, a ranking order of the top five features with the most significant contributions emerged: age, diarrhea, diabetes, hypertension, and BMI (Figure 3B). The average effect of age was positive or negative (±) 0.25, with reference to the baseline prediction of “survival” (0). Among the first five features with the highest importance scores, age, and BMI fell under the category of *“*patients” sociodemographic characteristics,' while diarrhea, diabetes, and hypertension were categorized as “pre-hospital comorbidities” (See Table 1).

SHAP served as an alternative to the model's “built-in” feature importance method based on “Gini impurity”. A notable distinction between these important measures was that the importance of the model-based “built-in” feature relied on decreased model performance. In contrast, SHAP provided insights into how individual features contributed to the model's output. Both plots were valuable for assessing feature importance, but they did not provide additional information beyond the importance itself, such as the direction of effect of each attribute of the variable (53). We examined the summary plot in the next section to address this limitation.

### 4.2. SHAP summary plot

**Figure 4:**
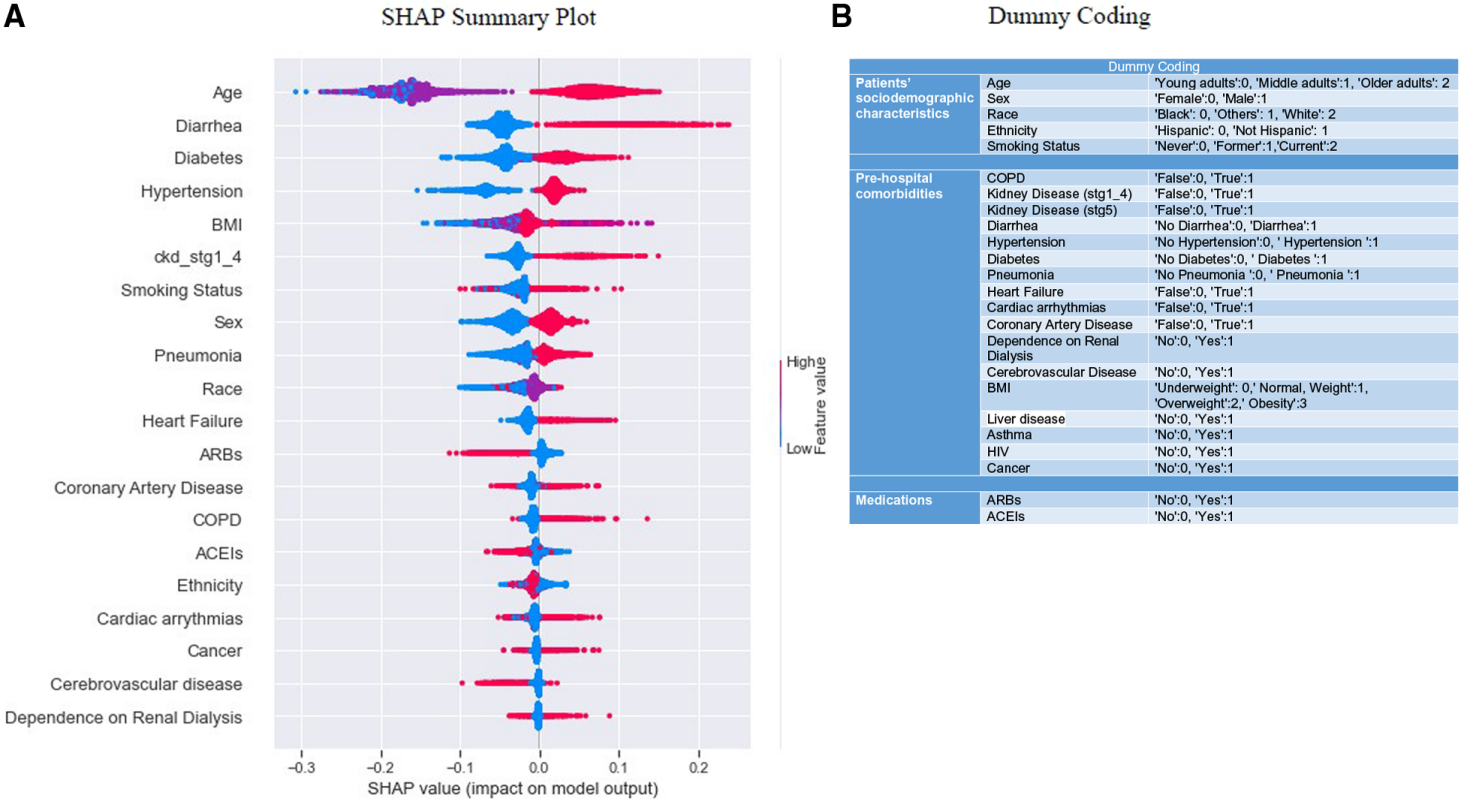
Summary plots for SHAP values. (**A**) indicates 20 features with the highest mean absolute SHAP values. Each point refers to each patient for the respective feature (row). A position of the point along the *x*-axis (i.e., SHAP value) represents the features’ influence on the model output for the explicit patient. A higher SHAP value has a higher impact on “mortality” relative to a lower SHAP value (54, 55). The features are arranged in order of importance on the *y*-axis, i.e., the more important features are placed at the top, given by their absolute Shapley values. Some outliers characterized by early stages of kidney disease, COPD, dependence on renal dialysis, etc., are also visible (green arrows). Positive SHAP values indicate an impact in predicting “mortality”, and negative SHAP values indicate an impact in predicting “survival” outcomes. (**B**) indicates the dummy coding, which could be used as a reference to compare the color coding [lower no represents a “cool” color, and higher no represents a “hot” color per category, as seen in (**A**)] of the SHAP beeswarm plot. (**A**) depicts “mortality” includes both linear-dominated relationships (in a box), such as diarrhea, sex, heart failure, etc., and non-linear-dominated relations, such as age, BMI, race, etc.

Beeswarm plot (Figure 4A) (54, 55), indicated the range across the SHAP value and pointed out the degradation probability, expressed as the logarithm of the odds (56). We could get a general idea of the directional impact of the features in relation to the distribution of “red” and “blue” dots. The colors of the points were related to the relative scaling of feature values. A SHAP value of “0” meant that the feature did nothing to move the decision away from the reference point “0”; thus, the feature had no contribution toward the decision of the model's prediction. The plot shows how features were highly influential, with strong “positive” or “negative” SHAP values for the predicted outcomes, and how the higher and lower values affected the result.

Values of each row to the right were “positive”, and those to the left had a “negative” impact on the model output. The “positive” and “negative” aspects were simply terms of the guideline and related to the direction in which model output was affected, which does not indicate how well the model performed. Along the *y*-axis, the features were arranged in decreasing order of importance. *X*-axis represented the SHAP value (e.g., the impact of the features on the model outcomes for the patients) (54). The color corresponded to the value of the function. As discussed before, the “red” color depicted a higher SHAP value of a feature that fell within the right distribution; on the other hand, “blue” color mapped a lower SHAP value of a feature that fell within the left distribution of the reference point “0” (32). For binary categorical variables (e.g., sex, diarrhea, diabetes, etc.), “red” meant “yes” and “blue” meant “no” depending on how they were coded (see “Dummy Coding”, Figure 4B). Each dot on the plot represented a single observation, vertically jittered when too close to each other. Figure 4A also revealed that “mortality” included both linear-dominated relationships (in a box, Figure 4A), such as diarrhea, sex, heart failure, etc., and non-linear-dominated relations, such as age, BMI, race, etc (56).

SHAP summary plot showed the top 20 features in ranking order and their impact on the “mortality” classification. For instance, the age variable had a high positive contribution to high and a low negative contribution to low values. This indicated that higher values for age led to higher predicted “mortality”, i.e., ages above 65 years old (“older adults”), and contributed the most to predicting death. Medicines (ARBs, ACEIs) appeared to have a reverse relationship. Using hypertension medications had a high “negative” contribution to “mortality”; while not using them had a high “positive” contribution to “mortality”. We also saw that no occurrence of diarrhea significantly reduced predicted “mortality” (“blue” dots), but the rise (extended “red” dots) was more significant than the drop, i.e., the larger values for this feature were associated with higher SHAP values.

From the SHAP summary plot, large values of BMI; i.e., “obese” and “overweight” contributed to the probability of belonging to one class (“mortality” or “survival”) and, in select cases, to another class (See the overlapping of “red” and “pink” colors in Figure 4A). We made this assertion based on the understanding that the impact of the feature's value depended on the entire sample. This is why we observed some “red” dots on the left side and some “blue” dots on the right side of the reference point “0”. (54). Effectively, SHAP showed us the global contribution by utilizing the feature importance and the local contribution for each feature instance through scattering the beeswarm plot.

### 4.3. Model explanation

**Figure 5:**
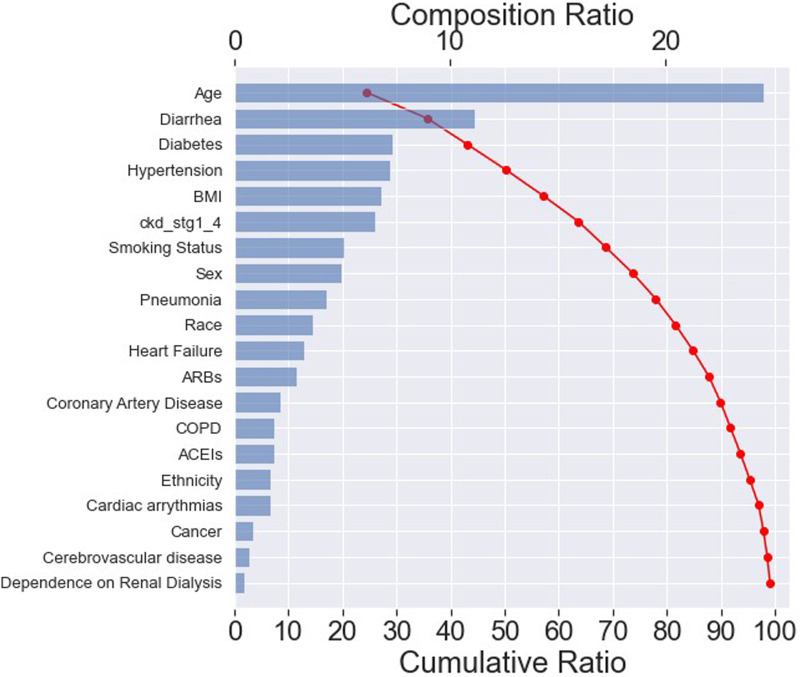
SHAP waterfall plot. The importance of each feature for predicting “mortality” in an RF classifier. The most important feature is age, resulting in a composition ratio of 20% (*x*-axis, top). The top 15 variables accumulate 95% of the model's cumulative ratio (*x*-axis, below). A cumulative ratio combines a composition ratio of 2 or more variables.

The effect of input variables on predicting the RF classifier for “mortality” was explored in more detail with the SHAP tool, as illustrated in the SHAP waterfall plot (Figure 5). The compositional ratio was estimated as the mean of absolute Shapley values per feature across the data (*x*-axis, top). The input variables were ordered according to their importance—the higher the mean SHAP value, the greater the importance of the variable (32).

The plot indicated that the top 15 features accounted for approximately 95% of the model's interpretation (*x*-axis, below). Furthermore, these top 20 features collectively contributed to nearly 100% of the model's interpretation. Among the top 15 most important features, 5 belonged to the “patients” sociodemographic characteristics', 8 pertained to “pre-hospital comorbidities”, and 2 were related to “medications”, as depicted in Table 1.

These findings suggested that the model could effectively capture the features within each category, with a primary emphasis on the “pre-hospital comorbidities”. As a result, it exhibited the ability to predict “mortality” accurately while maintaining transparency and reliability.

## 5. Discussion

Model performance of this study was aligned with the other **M**achine **L**earning (ML) tools utilized in various healthcare domains (7, 41, 44, 50, 54, 57, 58). It demonstrated the beneficial capabilities in predicting the severity of illness related to COVID-19, where disease progression remains unpredictable, both at the beginning of the virologic phase and the end of the inflammatory phase.

This study employed a traditional ML classifier to investigate the clinical variables associated with COVID-19 “mortality” among hospitalized patients in Southern Florida. To the best of our knowledge, this study contributed to one of the initial predictive analyses that applied AI/ML techniques to COVID-19 data using a vast sample from South Florida. Using the current ML approach, we confirmed the reported factors and expanded knowledge predicting the “mortality” outcome for “5,371” hospitalized patients with COVID-19.

In this exploratory data analysis, we trained an RF-based classification model to predict the prevalence of COVID-19 “mortality” using patients' pre-existing health conditions, such as “patients” sociodemographic characteristics', “pre-hospital comorbidities”, and “medications”. We demonstrated the utility of both the model's “built-in” technique and SHAP analysis to enhance the interpretation of factors associated with the “mortality” of in-patients with COVID-19.

For this study, 24 independent variables were selected to train a predictive model based on the learning from eHR data to analyze the data of the prospective cohort. Despite extensive work on optimizing feature importance, the AUC yielded 0.76 for predicting “mortality” in the test dataset where AUC scores were reported, in the previous studies, as 0.74 for predicting ICU admission and 0.83 for “mortality” (22). The model performance might differ due to the availability of a smaller number of features and populations.

As already mentioned, the training dataset was imbalanced initially, which referred to datasets where the target class had an uneven distribution of observations, i.e., the “survival” class had a very high number of observations, and “mortality” had a very low number of observations. Imbalanced classifications represented a challenge for predictive modeling since most of the ML algorithms used for classification have been built based on the assumption of an equal distribution for each class. As a result, models had poor predictive performance, particularly for the minority class. However, the minority class was more important; consequently, the problem was more sensitive to the misclassification of the minority class than the majority class. We did not balance the test data set for the model evaluation because we knew the real-world data set could be imbalanced in the specific scenario.

The SHAP functions for each variable indicated the individual feature's influence on the model for predicting “mortality”. Our study identified age as the most important clinical feature in COVID-19 patients, followed by diarrhea, diabetes, hypertension, BMI, CKD stages 1–4, smoking status, sex, pneumonia, and race in ranking order for “10” key factors. These findings aligned with previous studies regarding clinical features and the frequency of comorbidities in patients with COVID-19. Consistent with previous reports, advanced age emerged as the most significant predictor of severe outcomes (48, 55, 59–63). Male sex was identified as a high-risk factor in in-patients with COVID-19 (15, 16, 24, 59–62, 64). Our analyses also suggested a higher frequency of “mortality” from COVID-19 infection among “Whites” and “Hispanic populations.”

This study evaluated the role of patients’ immunocompromised status in exacerbating the severity of COVID-19, leading to death. It demonstrated a coherent association between COVID-19-related “mortality” and the underlying cause of immune suppression, such as diarrhea, diabetes, and hypertension. The study also reported that regular use of medicines, such as ARBS and ACEs, to treat high blood and heart failure could reduce the high incidence of “mortality” (65–67).

As can be noticed, these key features were slightly different from those selected by the statistical methods. According to statistical analysis, the following features were statistically significant. i.e., age, sex, smoking status, diabetes, hypertension, CKD stages 1–4, heart failure, pneumonia, ARBs, ACEIs, and diarrhea. However, of the above features, only 3 features (heart failure and medications such as ARBs and ACEIs) were not included in the top 10 features of the SHAP analysis. Similarly, race was included in the top 10 features of SHAP analysis but not statistically significant. The purpose of a statistical method is to find and explain the relationships between variables; alternatively, the ML model works on lesser assumptions and caters to patterns of data without an *a priori* understanding of the relationship between data and results (68). Thus, the ML model would demonstrate improved predictive potency in clinical settings.

### 5.1. Future work

The existing model is currently undergoing further refinement to enhance its accuracy. We have trained the model using a dataset of over 5,000 patients with COVID-19 in South Florida to predict “mortality” and assess disease severity based on patient characteristics. Our team is actively refining the algorithm and incorporating additional data points from diverse socio-demographic backgrounds to improve the model's robustness and enhance its ability to forecast disease outcomes accurately. As such, this work establishes the foundation for future research intending to forecast patient responses to treatments across different levels of disease severity and examine health disparities and patient conditions to enhance healthcare in a broader context. For example, future work can continue to utilize the same cohort, independent variables, and tree-based model design, such as Decision Trees and RF classifier, but focus on different outcome variables (e.g., ICU, MICU, and Mechanical Ventilation). By comparing different outcome variables, the intent can be to identify common features and assess the combined effects of two or more key features on the outcomes.

Furthermore, this research aims to provide comprehensive reports in a visual format, such as descriptive charts, tables, and plots, to offer valuable insights into various health issues. These reports will benefit clinicians and patients, as well as enable them to gain a deeper understanding of the health problems at hand and make informed decisions based on the available information.

### 5.2. Limitations

It is important to acknowledge that the data used in this study were derived from medical records, which had limitations and “built-in” constraints regarding the candidate variables. Additionally, symptoms were present before arrival at the hospital, but it was not determined at that time whether they were related to COVID-19. These limitations could have affected the strict adherence to the data collection protocol, potentially leading to the overestimation or underestimation of comorbidity and its impact on COVID-19 exacerbation. As a result, there is a possibility of false outcomes or errors. Additionally, due to the longitudinal nature of the study paradigm, patient selection bias and incomplete, missing, or inaccurate data were inevitable. Furthermore, it is crucial to highlight that the data used in this study did not account for the vaccination status of the patients. Considering the impact of vaccinations on COVID-19 outcomes is important when interpreting the results.

Moreover, it is important to note that the overall performance of the model does not indicate the precise risk probability determined by the algorithm at each time frame. Clinicians should not solely rely on the punctual predictability score as a diagnosis but rather assess the trend measurement by integrating the data within the context of clinical judgment.

## 6. Conclusion

This approach has the potential to offer practical clinical value to the healthcare system by utilizing a straightforward and objective tool, such as AI-based feature analysis, to stratify patients based on risk. This enables clinicians to triage patients with COVID-19 more efficiently, particularly in situations where resources may be limited. Additionally, this work provides insights to frontline workers by identifying the key contributors to COVID-19-related death in the South Florida region. Consequently, it holds the potential to aid in controlling the “mortality” rate associated with this disease. Moreover, by identifying comorbidities in advance, proactive healthcare activities can be initiated prior to hospital care.

We also noted that with adequate training, the model could effectively classify “mortality” and other disease severities, such as ICU admission and mechanical ventilation, using similar data and tools. Furthermore, as the dataset continues to grow, it will be possible to gain improved insights into the relationships between comorbidities and COVID-19 illness. In the future, the predictive model's capabilities will be established using a global-scale dataset.

Our hope is that this work will encourage the healthcare sector to integrate such explanatory tools into their workflow, thereby enhancing personalized healthcare. Subsequently, computer-aided platforms can utilize novel AI architecture to generate insights into the enduring clinical impact and discover better solutions to combat the ongoing pandemic.

## Data Availability

The data analyzed in this study is subject to the following licenses/restrictions: This dataset is provided by the South Florida Memorial Health Care System for analysis. We can not make this publicly available without their permission. You can contact the corresponding author to send a request to the contact person at South Florida Memorial Health Care System to get permission to access the data.
